# Role of FAD-I in Fusobacterial Interspecies Interaction and Biofilm Formation

**DOI:** 10.3390/microorganisms8010070

**Published:** 2020-01-02

**Authors:** Bhumika Shokeen, Jane Park, Emily Duong, Sonam Rambhia, Manash Paul, Aaron Weinberg, Wenyuan Shi, Renate Lux

**Affiliations:** 1 Section of Periodontics, Division of Constitutive & Regenerative Sciences, UCLA School of Dentistry, Los Angeles, CA 90095, USA; 2 David Geffen School of Medicine, UCLA, Los Angeles, CA 90095, USA; 3 Department of Biological Sciences, Case Western Reserve University, Cleveland, OH 44106-4905, USA; 4 The Forsyth Institute, Cambridge, MA 02142, USA

**Keywords:** *Fusobacterium nucleatum*, *fad-I*, RadD, interspecies interaction, biofilm

## Abstract

RadD, a major adhesin of oral fusobacteria, is part of a four-gene operon encoding the small lipoprotein FAD-I and two currently uncharacterized small proteins encoded by the *rapA* and *rapB* genes. Previously, we described a role for FAD-I in the induction of human B-defensin 2 (hBD2) upon contact with oral epithelial cells. Here, we investigated potential roles for *fad-I*, *rapA*, and *rapB* in interspecies interaction and biofilm formation. Gene inactivation mutants were generated for each of these genes in the *nucleatum* and *polymorphum* subspecies of *Fusobacterium nucleatum* and characterized for their adherence to partner species, biofilm formation, and operon transcription. Binding to *Streptococcus gordonii* was increased in all mutant strains with Δ*fad-I* having the most significant effect. This increased adherence was directly proportional to elevated *radD* transcript levels and resulted in significantly different architecture and height of the biofilms formed by Δ*fad-I* and *S. gordonii* compared to the wild-type parent. In conclusion, FAD-I is important for fusobacterial interspecies interaction as its lack leads to increased production of the RadD adhesin suggesting a role of FAD-I in its regulation. This regulatory effect does not require the presence of functional RadD.

## 1. Introduction

Bacterial adhesion is an essential process in inter-species interaction that ultimately enables the development and maturation of multi-species biofilm. Adhesion has been observed among all the genetically distinct bacteria isolated from a number of human sites including the gut, urogenital tract [1], and the oral cavity [2]. It is mainly mediated by the interaction of bacterial adhesins to a specific co-receptor in the partner species [3]. Many adhesins are subject to complex and coordinated regulatory processes in response to quorum sensing, bacterial stress, and host susceptibility, which consequently impacts the development of multi-species biofilms [4,5,6,7,8]. Biofilms of the oral cavity are one of the most characterized biofilms as they are easily accessible and the main cause for the progression of diseases like periodontitis and caries [9].

*Fusobacterium nucleatum*, an anaerobic, gram-negative commensal and opportunistic pathogen, is one of the most abundant microorganisms of the oral cavity [10,11,12,13]. It is an important species in dental biofilm ecology as it interacts with both early and late colonizers. The adhesive nature of *Fusobacterium* can be attributed to its repertoire of adhesins enabling the binding to various salivary proteins, other microorganisms, and host substrata [14,15,16,17,18]. One fusobacterial adhesin in particular, RadD, was identified as the main adhesin to mediate attachment to a number of gram-positive early colonizers [15] and supports fusobacterial adherence to certain isolates of the periodontal pathogen *Porphyromonas gingivalis* [19]. The RadD adhesin has also been implicated in the induction of cell death in human lymphocytes [20], indicating that this adhesin may be critical in the establishment of disease. Sequence and transcriptional analyses revealed that RadD is encoded by the last gene of a four-gene operon, which is conserved across all four subspecies of *F. nucleatum* [15]. The gene directly upstream of *radD*, previously described as *fad-I*, encodes the lipoprotein FAD-I, which is characterized by its ability to induce human β-defensin 2 (hBD-2) in oral epithelial cells in a subspecies-dependent manner [21,22]. FAD-I of *F. nucleatum* ssp *nucleatum*, type strains ATCC 25586 and ATCC 23726, induce expression of hBD-2, while FAD-I of *F. nucleatum* ssp *polymorphum*, type strain ATCC 10953, fails to do so. This differential antimicrobial peptide induction could have a profound influence on oral community composition. No function has been identified for the two other proteins encoded by the additional genes in the operon, which we denominated as RapA and RapB (RadD-associated proteins). While a recent study highlights the similarity of RapA to the fusobacterial adhesin FadA and suggests it to be FadA2, functional characterization is still missing [23].

In the present study, we performed a comprehensive investigation of the role of the genes encoded by the *radD*-containing four-gene operon in interspecies interaction of the *F. nucleatum* ssp *nucleatum* type strain ATCC 23726 *and F. nucleatum* ssp *polymorphum* type strain ATCC 10953. Our studies revealed that lack of *fad-I* led to increased binding of fusobacteria to *Streptococcus gordonii* by triggering significant overexpression of *radD*, which resulted in an increase in thickness and density of the biofilm formed with this important partner species. Furthermore, we demonstrated that the presence of the RadD adhesin is not needed for FAD-I-mediated regulation *of radD* expression and that the binding to *S. gordonii* leads to suppression of *radD* transcription in a FAD-I-independent manner.

## 2. Materials and Methods

### 2.1. Bacterial Strains and Culture Conditions

*F. nucleatum* ssp *nucleatum* strain ATCC 23726, *F. nucleatum* ssp *polymorphum* strain ATCC 10953, and their respective mutant derivatives generated in this study (Table 1) were maintained on Columbia agar supplemented with 5% sheep blood or in Columbia broth (CB) (Difco, Detroit, MI) under anaerobic conditions (10% H_2_, 10% CO_2_, 80% N_2_) at 37 °C. Strain *S. gordonii* Challis DL1 and its mCherry-expressing derivative [24] were maintained on Brain Heart Infusion) (BHI) agar or BHI broth (Difco, Detroit, MI) under anaerobic conditions (10% H_2_, 10% CO_2_, 80% N_2_) at 37 °C. Thiamphenicol at 5 μg mL^−1^ and clindamycin at 1 μg mL^−1^ (MP Biomedicals, Irvine, CA) were used for the selection and maintenance of strains possessing the *catP* and *ermB* cassette, respectively.

### 2.2. Mutant Strain and Plasmid Construction

#### 2.2.1. Allelic Exchange Mutagenesis

For the generation of mutants, allelic exchange mutagenesis was used to replace the gene of interest with the *catP* gene that confers thiamphenicol resistance in transformable fusobacteria. Briefly, the constructs were generated by fusing upstream (1 KB) and downstream (1 KB) regions of gene of interest to either end of the *catP* gene using the primers listed in Supplementary Table S1. The *catP* gene for each construct was amplified from pHS31 [26]. The primers contained an overlap of 25–30 base pairs to allow fusion in PCR reactions and the fusion PCR was carried out with Phusion HF DNA polymerase (NEB) as described [28]. The fusion product was cloned into pCR-Blunt II TOPO (Invitrogen) and transformed into One Shot TOP10 competent *E. coli* cells according to manufacturer’s protocols to generate the respective plasmids used for gene replacement (Table 1). The plasmid DNA of the recombinants were isolated with the Qiagen Mini prep kit (Qiagen, Hilden, Germany) and the presence of construct was confirmed by restriction digestion and sequencing. The construct was further subcloned in the suicide vector pHS31 as follows: both the fusion construct and pHS31 were digested with EcoRI/BamHI and purified prior to ligation (Supplementary Figure S1) and transformation into *Escherichia coli*. After confirmation of the integration plasmid by restriction analysis and sequencing, purified plasmids were electroporated into the fusobacterial strains used in this study to generate the respective derivatives lacking target genes according to previously described protocols [29]. The transformed products were plated on selective media containing 5 μg mL^−1^ thiamphenicol. The genomic DNA of the colonies obtained after transformation was isolated using GENelute kit (SIGMA) and analyzed by PCR with internal primers for presence of *catP* gene and absence of the respective gene.

#### 2.2.2. Complementation

For generation of the strain complementing lack of *fad-I* in the Δ*fad-I* mutant of *F. nucleatum* ssp *nucleatum* ATCC 23726, the previously described plasmid pBS5 [22] was transformed into the Δ*fad-I* mutant of ATCC 23726 as described [29].

### 2.3. Coaggregation Assay

Coaggregation assays were performed according to previously published protocols [15,19] in coaggregation buffer (CAB; 150 mM NaCl, 1 mM Tris, 0.1 mM CaCl_2_, 0.1 mM MgCl_2_ H_2_O [pH 7.5]). Briefly, cells were pelleted and resuspended in CAB to a final concentration of 2 × 10^9^ cells (OD_600_ of 2). Suspensions of strains to be examined for coaggregation were combined with an equal volume of a test strain adjusted to the same cellular concentration in CAB to a total volume of 400 μL in a reaction tube. The optical densities of reaction mixtures were obtained spectrophotometrically immediately after addition of the second partner strain and vortexing (OD_t_ = 0 min). After 10 min of incubation, reaction mixtures were centrifuged at low speed (100× *g* for 1 min) to pellet coaggregated cells, while leaving non-aggregated bacteria in suspension. Optical densities of the supernatants were measured after the 10 min incubation (OD_t_ = 10 min) in order to quantify coaggregation. The level of inter-species binding is expressed as % coaggregation, which refers to the proportion of cells that fall out of the original cell suspension due to aggregate formation between partner species. The % coaggregation of the test reactions were calculated as (OD_t_ = 0 min − OD_t_ = 10 min)/(OD_t_ = 0 min). These values were averaged across at least three independent experiments and represented as percentages.

### 2.4. Co-Incubation of F. Nucleatum with Partner Species

Cells were grown to mid-log phase and 1 mL of 10^9^ cells (OD_600_ = 1) of *F. nucleatum* or its mutant derivatives were added to sterile 2 mL microcentrifuge tubes with 1 mL of 10^9^ cells of *S. gordonii* (OD_600_ = 1). Control tubes containing 1 mL of 10^9^ cells of *F. nucleatum* or its mutant derivatives alone were also prepared. Cells were centrifuged for 5 min at 8000× *g*, the supernatant was discarded, and replaced with 1 mL of CB for *F. nucleatum alone* tubes, 2 mL of CB for tubes with *F. nucleatum* ATCC 23726 and *S. gordonii.* The pellets were incubated anaerobically (10% H_2_, 10% CO_2_, 80% N_2_) at 37 °C for 30 min. Post incubation, the supernatant was removed after a quick centrifugation and the cell pellets were stored in −80 °C.

### 2.5. Nucleic Acid (DNA and RNA) Isolation and cDNA Generation

Genomic DNA was isolated using the GenElute Bacterial Genomic DNA Kit (Sigma-Aldrich, St. Louis, MO, USA), according to the manufacturer’s instructions. Total RNA was extracted from cells using the PureLink RNA Mini Kit (Thermo Fisher Scientific, Waltham, MA, USA), followed by a TURBO DNA-free DNase Treatment (Thermo Fisher Scientific, Waltham, MA, USA), prior to cleaning and concentration with the RNA Clean & Concentrator (Zymo Research, Irvine, CA, USA). One microgram of total RNA was used for cDNA synthesis with the SuperScript III First-Strand Synthesis System (Thermo Fisher Scientific, Waltham, MA, USA) according to the manufacturer’s protocol.

### 2.6. Quantitative (Real-Time) Polymerase Chain Reaction

Gene-specific primers (Table 2) were used to amplify transcript regions for signal detection by qPCR with the iCycler Thermal Cycler (Bio-Rad, Hercules, CA, USA) in a total reaction volume of 20 μL containing 10 μL of 2X iQ SYBR Green Supermix (Bio-Rad, Hercules, CA, USA), 0.5 μM each of forward and reverse primers, and 1 μL of 1:10 diluted cDNA.

Amplification and detection were carried out in 96-well optical plates (Thermo Fisher Scientific, Waltham, MA). Each qPCR run was performed with an initial incubation of 10 min at 95 °C followed by 40 cycles of denaturing at 95 °C for 15 s and annealing and elongation at 60 °C for 1 min. After the 40 cycles of amplification, an additional denaturing step was performed at 95 °C for 1 min followed by annealing and elongation at 60 °C for 1 min. A melting curve analysis was completed after each run. Three independent qPCR runs were performed with three technical replicates for each sample to assess reproducibility and inter-run variability. Following amplification, relative expression levels between samples were calculated as fold changes normalized to *rpoB* reference gene amplification.

### 2.7. Biofilm Growth

#### 2.7.1. Crystal Violet Assay

Dual-species biofilms of *F. nucleatum* ssp *nucleatum* (wild-type and its mutant strains) with *S. gordonii* were grown in sterile 48-well culture plates (Thermo Fisher Scientific, Waltham, MA, USA). Briefly, fusobacterial and *S. gordonii* cells were diluted from overnight cultures to 2 × 10^8^ and 1 × 10^5^ cells, respectively, into 50% SHI medium [30] and incubated under anaerobic conditions (10% H_2_, 10% CO_2_, 90% N_2_) for 20 h. The biomass of the biofilms was evaluated by crystal violet (CV) staining according to published procedures [31]. In brief, supernatants were removed from each well and rinsed once with 500 μL of sterile phosphate-buffered saline (PBS). Plates were inverted and dried. Next, attached bacteria were fixed at room temperature for 15 min by adding 500 μL of methanol into each well. The biofilms were then stained with a 500 μL aqueous solution of 0.5% crystal violet (Thermo Fisher Scientific, Waltham, MA, USA) for 15 min at room temperature, followed by careful rinsing with Millipore water until there was no visible trace of the stain left. Bound stain was extracted by adding 500 μL of 95% ethanol. The optical density of each well was measured at 570 nm and was represented as relative to negative control wells that only contained SHI medium. All wild-type and mutant combinations as well as mono-species control biofilms were grown in at least three biological replicates.

#### 2.7.2. Confocal Microscopy

Dual-species biofilms of *F. nucleatum* ATCC 23726 and the mCherry-expressing derivative of *S. gordonii* challis DL1 [24] were grown in 8-well chambers on optical plastic slides (Thermo Fisher Scientific, Waltham, MA), which were coated with microorganism-free pooled saliva and UV-sterilized for 1 h. Biofilms were inoculated and grown as described above for the crystal violet assay and washed with sterile PBS before adding 20 μM SYTO 9 (green) for visualization by confocal microscopy with an LSM-780 confocal microscope (Zeiss, Germany). A Zeiss plan-Apochromat 63× oil immersion objective was used for image acquisition. SYTO 9 fluorescence was imaged using a 488 nm laser with a 500 to 530 nm emission range capture and then pseudo-colored as blue. The mCherry-expressing *S. gordonii* in the biofilm was visualized using a 561 nm excitation laser in combination with a 600 to 650 nm emission range capture. Orthogonal sectioning of the z-stacks and height measurements were performed using the Zeiss Zen software. Multiple areas of the biofilm (n = 5) were imaged following a consistent x-y grid. Data from all 3 set of experiments were used to calculate the mean height of the biofilm. The height of the biofilm was determined using the ZEN Blue Image Analysis Module (Zeiss, Germany).

### 2.8. Statistical Analysis

Student’s *t*-test was performed to determine statistical significance using Excel (Microsoft, Seattle, WA, USA, Version 2016).

## 3. Results

In fusobacteria, including the genetically tractable strains of *F. nucleatum* ssp *nucleatum* ATCC 23726 and *F. nucleatum* ssp *polymorphum* ATCC 10953, the adhesin RadD is encoded by the last gene of a four-gene operon [15], comprised of the homologs of FN1529, FN1528, FN1527 (*fad-I*) [21], and FN1526 (*radD*). The corresponding homologs of these genes in the *nucleatum* subspecies ATCC 23726 are encoded by HMPREF0397_1642, HMPREF0397_1641, HMPREF0397_1640, and HMPREF0397_1639, respectively, and the open reading frames FNP_1046, FNP_1047, FNP_1048, and FNP_1049 in the *polymorphum* subspecies ATCC 10953 [32]. The genes encoded by homologs of FN1529 and FN1528 were previously unnamed and will be referred to in this study as *rapA (radD*-associated proteins A) and *rapB*, respectively. Based on the sequence similarity of RapA to the previously characterized FadA adhesin [18], *rapA* was also suggested to be named *fadA2* by a very recent bioinformatic study [23]. All the four genes of the operon are predicted to be associated with membrane with Predict Protein (www.predictprotein.com accessed on 10 October 2019). RapA (127aa) is predicted to be a member of the FadA family [18,23], while RapB (123aa) is predicted to be a lipoprotein. We previously identified FAD_I (129aa) as lipoprotein [21,22] and RadD (3546aa) as an autotransporter-like adhesin [15,20].

**Figure 1:**
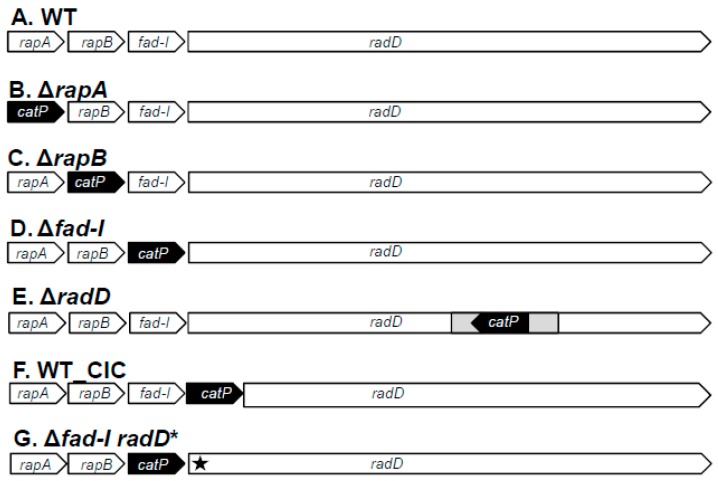
Schematic representation of gene inactivation mutants of the *radD*-encoding four-gene operon and controls (**A**) wild-type (WT), (**B**) Δ*rapA*, (**C**) Δ*rapB*, (**D**) Δ*fad-I*, (**E**) Δ*radD* (**F**), and wild-type-*catP* insertion control (WT_CIC) in *F. nucleatum* subspecies *nucleatum* and *polymorphum*. (**G**) Δfad-I radD*: *F. nucleatum ssp. nucleatum* ATCC 23726 *radD* frameshift mutant in the Δ*fad*-*I* background.

In our earlier studies, we inactivated *radD* of ATCC 23726 and ATCC 10953 as well as *fad-I* of ATCC 10953 [15,22,25] as part of their functional characterization. While we demonstrated a role for RadD as a major adhesin for interaction with streptococcal species, investigation of FAD_I was limited to its role in hBD2 induction in epithelial cells. For a comprehensive investigation of all genes encoded by the *radD*-containing four-gene operon in interspecies interaction, we created individual gene inactivation mutants in the *rapA*, *rapB*, and *fad-I* genes in *F. nucleatum* ssp *nucleatum* ATCC 23726 as well as *rapA* and *rapB* in *F. nucleatum* ssp *polymorphum* ATCC 10953 (Figure 1) with the approaches described in Material and Methods. As mentioned above, inactivation mutants of ATCC 23726 and ATCC 10953 lacking the remaining genes encoded by the *radD*-containing operon were generated in our previous studies [15,22,25]. We also constructed strain WT-CIC (*catP* Insertion Control) in which the *catP* resistance cassette was inserted between *fad-I* and *radD* as a control strain to address possible polar effects of *catP* insertion on gene expression (Figure 1).

### 3.1. Inactivation of radD-Operon Genes in F. Nucleatum Alters Coaggregation with S. Gordonii

**Figure 2:**
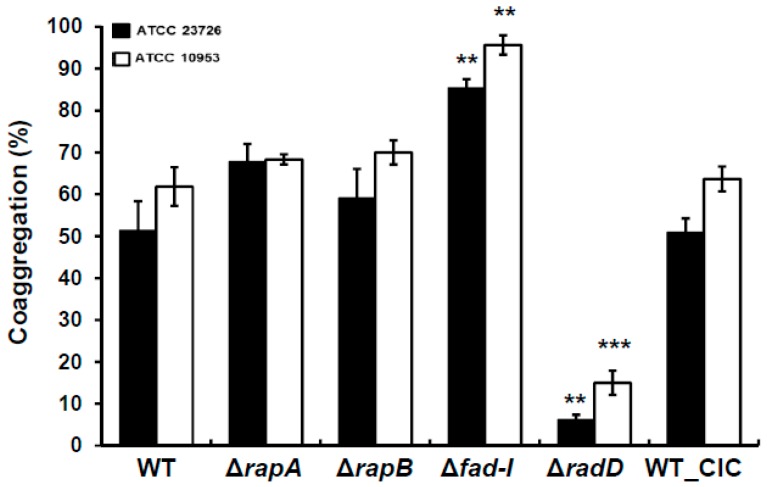
Quantitative Coaggregation of wild-type *F. nucleatum ssp. nucleatum* (ATCC 23726) and *F. nucleatum ssp. polymorphum* (ATCC 10953) and their various mutant derivatives: Δ*rapA*, Δ*rapB*, Δ*fad*-*I*, Δ*radD*, and control (WT_CIC) with *S. gordonii.* Data are represented as mean of percentage coaggregation and standard error of mean of three independent experiments. (** *p* ≤ 0.01 and *** *p* ≤ 0.001 compared to the wild-type control).

The Δ*rapA*, Δ*rapB*, Δ*fad-I*, and Δ*radD* mutant derivatives of both *F. nucleatum* ssp *nucleatum* and ssp *polymorphum* were subjected to coaggregation assays with *S. gordonii* to characterize their binding to this important partner species. Quantitative coaggregation assays revealed similar coaggregation behavior for the corresponding mutants of both fusobacterial subspecies with *S. gordonii* (Figure 2). Individual inactivation of the genes encoding the RapA and RapB proteins did not result in significant changes compared to the wild-type in either one of the two *F. nucleatum* subspecies tested. Specifically, coaggregation of *S. gordonii* with the Δ*rapA* mutants of *F. nucleatum* ssp *nucleatum* and *F. nucleatum* ssp *polymorphum* resulted in 67 ± 4% and 68 ± 1% coaggregation, respectively, while coaggregation with the Δ*rapB* derivatives was 59 ± 7% for *F. nucleatum* ssp *nucleatum* and 70 ± 3% for *F. nucleatum* ssp *polymorphum* compared to 51 ± 7% and 62 ± 4% for the corresponding wild-type strains. In contrast to the Δ*rapA* and Δ*rapB* mutants, the Δ*fad-I* mutants in both subspecies showed significantly increased levels of coaggregation compared to the wild-type (85 ± 2% for *F. nucleatum* ssp *nucleatum* 23726 and 96 ± 2% for *F. nucleatum* ssp *polymorphum* 10953) (Figure 2). Consistent with our previous reports, the *radD* mutants for both strains displayed a coaggregation deficient phenotype (*F. nucleatum* ssp *nucleatum*, 6 ± 1% coaggregation and *F. nucleatum* ssp *polymorphum*, 15 ± 3% coaggregation), confirming RadD as a major adhesin in the interaction with *S. gordonii*. As expected, the WT-CIC strain of both *F. nucleatum* ssp *nucleatum* and *F. nucleatum* ssp *polymorphum* exhibited coaggregation levels similar to their wild-type parents with 50 ± 3% and 63 ± 3%, respectively.

### 3.2. Enhanced Coaggregation in Δfad-I Mutant Correlates with Increased RadD Expression

**Figure 3:**
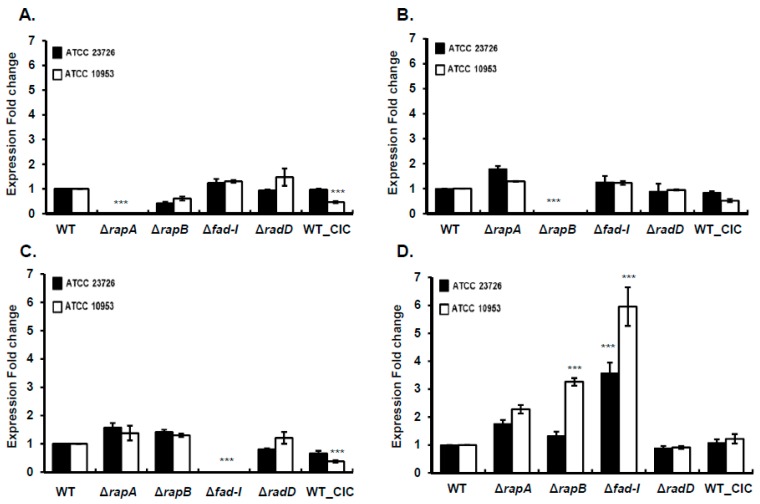
Transcriptional analysis of *radD*-operon genes in wild-type *F. nucleatum ssp. nucleatum* (ATCC 23726) and *F. nucleatum ssp. polymorphum* (ATCC 10953) and their mutant derivatives. Expression fold changes compared to wild-type are shown for (**A**) *rapA*, (**B**) *rapB*, (**C**) *fad-I*, and (**D**) *radD* for mutant derivatives and the WT_CIC control strains. Data are presented as the mean and standard error of mean of three independent experiments. (*** represents *p* ≤ 0.001 compared to the wild-type control).

Next, we employed qPCR to determine how the lack of RapA, RapB, FAD-I, and RadD affects *rapA*, *rapB*, *fad-I*, and *radD* expression (Figure 3). As expected, no transcripts were detected in the Δ*rapA*, Δ*rapB*, and Δ*fad-I* mutant derivatives for genes that were replaced by *catP* gene (Figure 1). However, *radD* transcript levels similar to wild-type can be detected in the *radD* mutants of both *F. nucleatum* ssp *nucleatum* and *F. nucleatum* ssp *polymorphum* by using qPCR primer designed upstream of the insertion. Even though some of the differences compared to wild-type gene expression were statistically significant due to the low data variability, they mostly did not exceed or even come close to two-fold changes in expression levels. This included *rapA* expression (Figure 3A) in Fnn_WT_CIC (0.97 ± 0.05), Fnp_Δ*rapB* (1.3 ± 0.14), Fnn_Δ*fad-I* (1.24 ± 0.29), Fnp_Δ*fad-I* (1.30 ± 0.95), Fnn_Δ*radD* (0.94 ± 0.06); Fnp_Δ*radD* 1.48 ± 0.60), *rapB* expression (Figure 3B) in Fnn_WT_CIC (0.84 ± 0.09), Fnp_WT_CIC (0.52 ± 0.11), Fnn_Δ*rapA* (1.92 ± 0.43), Fnp_Δ*rapA* (1.29 ± 0.02), Fnn_Δ*fad-I* (1.27 ± 0.42), Fnp_Δ*fad-I* (1.23 ± 0.13), Fnn_Δ*radD* (0.90 ± 0.52), Fnp_Δ*radD* (0.95 ± 0.04), and *fad-I* expression (Figure 3C) in Fnn_Δ*rapA* (1.72 ± 0.49), Fnp_Δ*rapA* (1.38 ± 0.46), Fnn_Δ*rapB* (1.42 ± 0.15), Fnp_Δ*rapB* (1.69 ± 0.58), Fnn_Δ*radD* (0.80 ± 0.07), Fnp_Δ*radD* (1.21 ± 0.36), Fnn_WT_CIC (0.67 ± 0.16). Exceptions were somewhat reduced expression of *rapA* in Fnn_Δ*rapB* (0.41 ± 0.12-fold change) and Fnp_WT_CIC (0.47 ± 0.08 fold change) (Figure 3A), in addition to lower expression of *fad-I* in Fnp_WT_CIC (0.38 ± 0.08 fold change) (Figure 3C). In contrast to the mostly unaltered expression levels of *rapA*, *rapB*, and *fad-I*, *radD* expression levels were significantly (*p* ≤ 0.001) elevated by several-fold in some of the mutant derivatives and especially in the Δ*fad-I* mutant (Figure 3D). Interestingly, elevated *radD* transcript levels correlated with the observed significant increase in coaggregation with *S. gordonii* (Figure 2). While the enhancing effect on *radD* expression was limited in the *F. nucleatum* subspecies *nucleatum* to the Δ*fad-I* mutant (Fnp_Δ*rapA*—1.76 ± 0.23; Fnp_Δ*rapB*—1.33 ± 0.26; Δ*fad-I*—3.49 ± 0.79), in the *polymorphum* subspecies, the lack of either one of these genes resulted in increased *radD* transcript levels with Fnp_Δ*fad-I* exhibiting the largest effect (Fnp_Δ*rapA*—2.28 ± 0.27; Fnp_Δ*rapB*—3.26 ± 0.23; Fnp_Δ*fad-I*—5.95 ± 1.20). Lack of the RadD protein does not seem to affect the expression of the encoding gene as *radD* transcript levels were similar to wild-type for Fnn_Δ*radD* (0.89 ± 0.13) and Fnp_Δ*radD* (0.91 ± 0.09). Furthermore, *radD* expression was not elevated in Fnn_WT_CIC (1.06 ± 0.20) and Fnp_WT_CIC (1.22 ± 0.29), which largely rules out polar effects of *catP* insertion upstream of *radD* as a cause for the observed altered expression.

Since lack of *fad-I* produced the strongest phenotype in both subspecies investigated in this study, we constructed an additional mutant strain, Fnp_Δ*fad*-I*, to distinguish if lack of the *fad-I* gene encoding sequence in the gene replacement mutants or the lack of the FAD-I protein led to the observed increase in *radD* expression. Strain Fnp_Δ*fad*-I* contains the full *fad-I* gene sequence but does not produce the FAD-I protein due to a mutated translation start codon (TGA was mutated to AAT). The Fnp_Δ*fad*-I* mutant strain displayed a very similar phenotype to the Fnp_Δ*fad*-*I* derivative with significantly increased *radD* expression and enhanced coaggregation with *S. gordonii* (Supplementary Figure S2). However, the Fnp_Δ*fad*-I* mutant was unstable with a strong tendency to revert to a start codon and was therefore excluded from further investigation.

### 3.3. Plasmid-Based fad-I Complemention Reduced Coaggregation and radD Transcript Levels in Fusobacterial Strains Lacking fad-I

**Figure 4:**
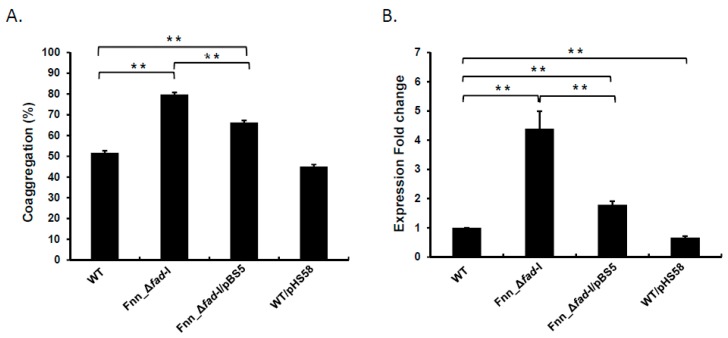
Characterization of the *fad*-*I* complement of *F. nucleatum ssp*. *nucleatum.* (**A**) Quantitative coaggregation of the mutant derivatives of wild-type ATCC 23726 (WT), Fnn_Δ*fad*-I, and Fnn_Δ*fad*-I/pBS5 with *S. gordonii* is shown as percentage coaggregation. (**B**) Transcriptional levels of *radD* in the Fnn_Δ*fad*-I mutant and the Fnn_Δ*fad*-I/pBS5 complement along with a vector only control WT/pHS58 are shown as fold change in comparison to the wild-type parent ATCC 23726. All data are presented as the mean and standard error of mean of three independent experiments (** represents *p* ≤ 0.01).

Lack of *fad-I* exhibited the most significant increases in *radD* transcripts levels as well as coaggregation with *S. gordonii* in both fusobacterial subspecies investigated in this study. Strain Fnn_Δ*fad-I* carrying the *fad-I*-expressing plasmid pBS5 [22] and pHS58 as empty vector control were assessed for coaggregation with *S. gordonii* and *radD* expression. We observed a significant reduction in the coaggregation percentage of the Fnn_Δ*fad-I*/pBS5 (66.2 ± 2.4) complemented strain compared to the Fnn_Δ*fad-I* carrying the empty pHS58 vector control (79.7 ± 1.3), although it was still higher than the wild-type (51.6 ± 2.8) (Figure 4A). Similarly, *radD* transcript levels were significantly reduced from 4.4-fold in the Fnn_Δ*fad-I* mutant to 1.8-fold in the Fnn_Δ*fad-I*/pBS5 complement strain (Figure 4B). This reduction, however, was not to the same level as present in the parent strain *F. nucleatum* ssp *nucleatum* ATCC 23726, indicating that plasmid-based FAD-I production does not allow complementation to wild-type levels.

### 3.4. The Presence of the RadD Adhesin Is Not Required for Increased Expression of radD in Δfad-I Mutant

**Figure 5:**
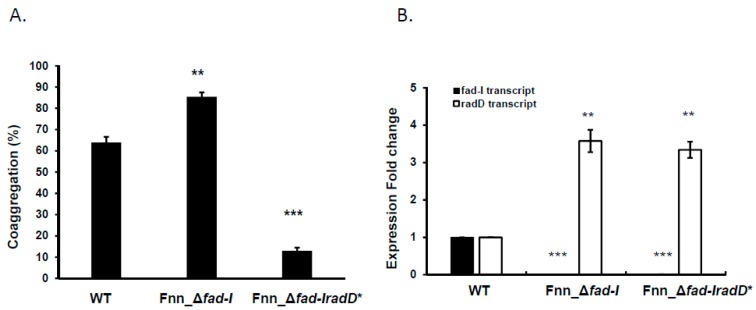
Quantitative coaggregation and transcriptional analysis of *F. nucleatum ssp*. *nucleatum*, Fnn_Δ*fad-I*, and Fnn_Δfad-IradD*. (**A**) Coaggregation of wild-type ATCC 23726 (WT), Fnn_Δ*fad-I*, and Fnn_Δfad-IradD* with *S. gordonii is* represented as mean of percentage coaggregation and standard error of mean of three independent experiments. (**B**) *fad-I and radD* transcript levels are represented as expression fold change compared to the wild-type parent ATCC 23726. The data represent the mean and standard error of mean of three independent experiments. (** represents *p* ≤ 0.01 compared to the WT control, *** represents *p* ≤ 0.001 than the WT).

To investigate if the observed *radD* regulation requires the RadD adhesin, we introduced a frameshift mutation in the N-terminal part of the *radD* gene in the Fnn_Δ*fad-I* mutant background to create a derivative in which *radD* is still expressed but not translated into a functional protein (Figure 1G). The resulting Fnn_Δfad-I radD* mutant strain was deficient in coaggregation with *S. gordonii* (13.0 ± 2.6% coaggregation compared to 64% for the wild-type parent control) (Figure 5A), confirming the absence of the RadD adhesin. Transcript levels for *radD* in the Fnn_Δfad-I radD* derivative remained elevated relative to wild-type (3.3 ± 0.2-fold increase) (Figure 5B) similar to our findings for the Fnn_Δ*fad-I* mutant (Figure 3D). These results indicate that the RadD adhesin is not needed for the increased *radD* transcripts in Δ*fad-I* mutant but mediates the enhanced coaggregation phenotype with the fusobacterial partner species *S. gordonii*.

### 3.5. Co-Incubation of F. Nucleatum ssp Nucleatum with S. Gordonii Reduces radD Transcript Levels

**Figure 6:**
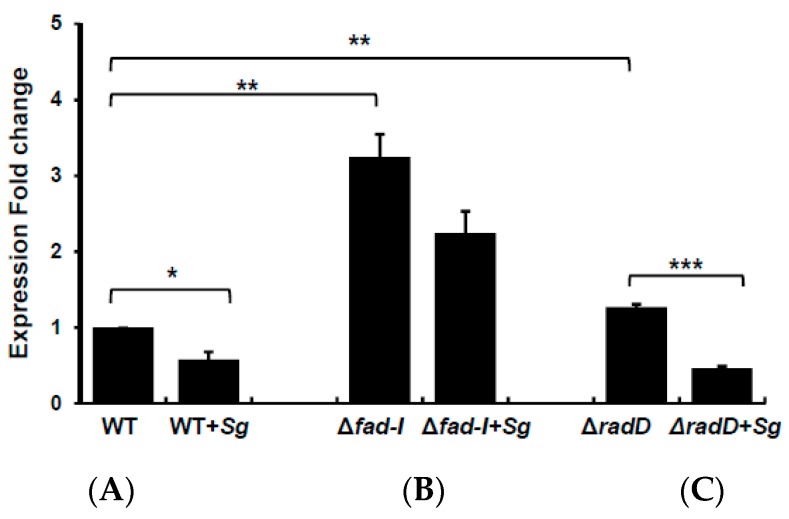
Transcriptional analysis of *radD* levels in the presence of *S. gordonii*. Presented are *radD* levels in (**A**) wild-type ATCC 23726 (WT), (**B**) Fnn_Δ*fad-I*, and (**C**) Fnn_Δ*radD* alone or in the presence of the partner species. Data are presented as the mean and standard error of mean of three independent experiments (* represents *p* ≤ 0.05, ** represents *p* ≤ 0.01, *** represents *p* ≤ 0.001).

*F. nucleatum* ssp *nucleatum* utilizes RadD as a major adhesin for binding to *S. gordonii* and other partner species. To examine if the presence of binding partner influences the regulation of this important adhesin on a transcriptional level, we determined *radD* expression in the presence and absence of *S. gordonii* (Figure 6). The presence of *S. gordonii* reduced *radD* transcript levels, when co-incubated with *F. nucleatum* ssp *nucleatum* ATCC 23726 within the first 30 min. The expression was significantly reduced by almost two-fold upon co-incubation with *S. gordonii*. The Δ*radD* derivative exhibited a similar significant reduction of *radD* expression in the presence of *S. gordonii* indicating that this regulatory effect does not require the adhesin. While a relative 1.5-fold reduction of *radD* transcript levels was observed in the Fnn_Δ*fad-I* mutant during co-incubation with *S. gordonii*, the difference was not significant and still resulted in a substantially higher (2.2-fold) expression of *radD* compared to the wild-type parent strain.

### 3.6. RadD Levels Are Important for Dual-Species Biofilm Formation of F. Nucleatum with S. Gordonii

**Figure 7:**
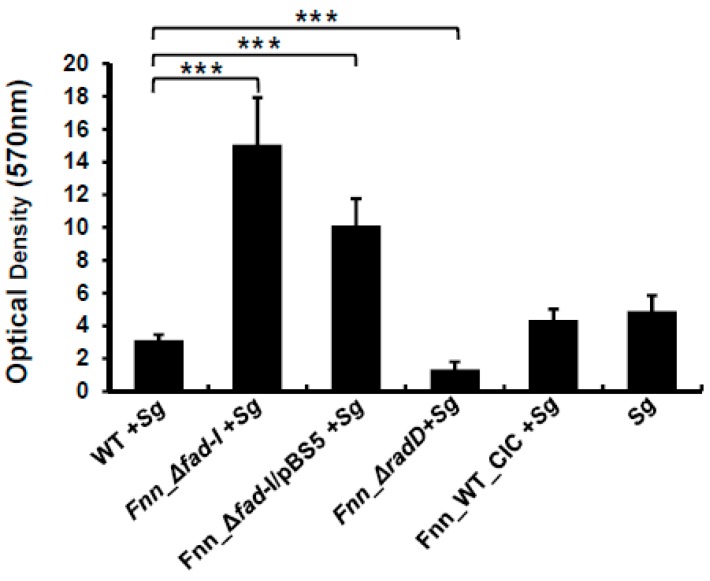
Biomass of dual-species biofilms formed by *F. nucleatum* ssp *nucleatum* ATCC 23726 and its *radD*-operon mutant derivatives with *S. gordonii*. The biomass was assessed via the crystal violet assay. Data are presented as the mean and standard error of mean of three independent experiments (*** represents *p* ≤ 0.001).

We further investigated if the increase in *radD* expression in the Δ*fad-I* strain affects biofilm development with *S. gordonii*. Staining with crystal violet revealed that the biomass of biofilms developed by *S. gordonii* alone (4.8 ± 0.9) was comparable to the biomass of dual-species biofilm formed by *F. nucleatum* ssp *nucleatum* ATCC 23736 (3.1 ± 0.3) and Fnn_WT_CIC (4.3 ± 0.7) in the presence of *S. gordonii* (Figure 7) under the growth conditions used. Not surprisingly, the corresponding *radD* mutant, which is defective in binding to streptococci [15] produced significantly reduced biomass with *S. gordonii* (1.9 ± 0.5). The Fnn_Δ*fad-I* mutant generated significantly more biomass (15.0 ± 2.8) during dual-species biofilm formation, consistent with the greatly increased attachment to *S. gordonii* (Figure 2). Similar to our findings above, in which we observed that plasmid-based complementation of lack of *fad-I* only partially reduces *radD* expression to wild-type levels, the complement strain phenotype exhibits a reduction in biomass production (10.1 ± 1.6) compared to the Fnn_Δ*fad-I* mutant strain but remains more than two-fold elevated over wild-type.

**Figure 8:**
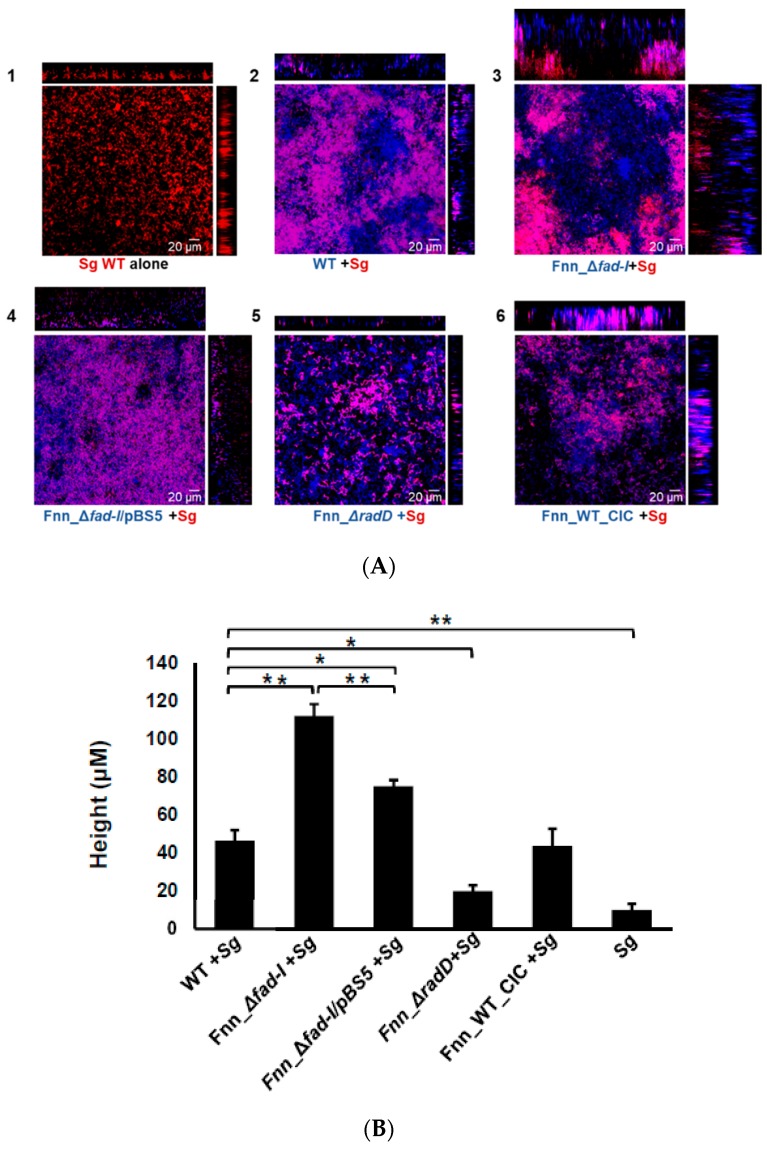
(**A**). Visualization of dual-species biofilm formed between wild-type and mutant strains of *F. nucleatum* ssp *nucleatum* ATCC 23726 and *S. gordonii* (mCherry) by CLSM. The biofilm was fluorescently labeled with SYTO9. The *S. gordonii* (Sg) cells constitutively express mCherry and appear red on the images. Wild-type (WT) *F. nucleatum* (Fn) and its mutants (*fad-I, fad-I/pBS*5, *radD*, WT-CIC) are stained by syto9-only which are pseudo-colored as blue in the Zen software. Association of *F. nucleatum* and *S. gordonii* in the biofilm is observed as purple color in the confocal images. Each image panel is represented by x-z axis view on top and y-z axis view on the right side of the x-y view. The various panels show the biofilm formed by: (**1**) *S. gordonii* (Sg) alone; (**2**) wild-type ATCC 23726 with *S. gordonii;* (**3**) Fnn_Δ*fad-I* with *S. gordonii;* (**4**) Fnn_Δ*fad-I*/pBS5 with *S. gordonii*; (**5**) Fnn_Δ*radD* with *S. gordonii;* (**6**) Fnn_WT_CIC with *S. gordonii*. (**B**) Comparison of the height of the dual species biofilm of the wild-type and mutant strains of *F. nucleatum* ssp *nucleatum* ATCC 23726 with *S. gordonii*, as observed from the confocal images. The data represents mean of the height and standard error of mean of biofilm as observed in three independent experiments with height measurements captured in five randomly chosen locations in each experiment (n = 15). The single species biofilm of *S. gordonii* is also included as control. (* represents *p* ≤ 0.05, ** represents *p* ≤ 0.01).

Next, we employed confocal microscopy to examine architectural features of dual-species biofilms by *S. gordonii* with *F. nucleatum* ssp *nucleatum* wild-type and its mutant derivatives investigated in this study (Figure 8A). Height measurements revealed that dual-species biofilms formed with *F. nucleatum* ssp *nucleatum* wild-type and the Fnn_WT_CIC control strain were significantly taller (46.30 ± 5.6 μm and 43.5 ± 9.10, respectively) compared to the mono-species biofilms formed by *S. gordonii* alone (9.9 ± 3.15 μm) (Figure 8B). This effect on biofilm height was greatly enhanced when *S. gordonii* was combined with the Fnn_Δ*fad-I* mutant instead of its wild-type parent. Biofilms with the *F. nucleatum* Δ*fad-I* (Fnn_Δ*fad-I)* mutant were significantly taller (112.16 ± 6.2 μm) confirming the importance of RadD levels in biofilm formation and development. Interestingly, the height of the biofilm formed by the *fad-I* complement strain (Fnn_Δ*fad-I*/pBS5) (75.1 ± 3.4), was reduced compared to the Fnn_Δ*fad-I* mutant, but still higher than the dual-species biofilm formed by the wild-type strain with *S. gordonii*. As expected, the Fnn_Δ*radD* mutant strain largely failed to integrate into the biofilm, which is reflected by a significantly shallower height (23.8 ± 5.75 μm) compared to those formed with the wild-type.

## 4. Discussions

In this study, we investigated the roles of the three small proteins (RapA, RapB, FAD-I) encoded upstream of the fusobacterial RadD adhesin. We previously identified RadD as a major adhesin in interspecies interaction with gram-positive species such as oral streptococci [15] in addition to its function in apoptosis induction in lymphocytes [20] and strain-specific fusobacterial binding to *P. gingivalis* [19]. The work presented here led to the discovery that the lipoprotein FAD-I, which is part of the *radD*-containing operon, controls interspecies interaction via regulation of *radD* expression in a RadD-independent manner.

The genes encoding RapA, RapB, and FAD-I were systematically inactivated in the *nucleatum* and *polymorphum* subspecies of *F. nucleatum* and the resulting mutant strains were characterized regarding their effects on interspecies interaction with the important fusobacterial partner strain *S. gordonii*. While lack of *rapA* and *rapB* did not have a consistent significant impact on *F. nucleatum* behavior in both subspecies tested, lack of *fad-I* produced a striking phenotype characterized by a significant increase of coaggregation as well as biofilm formation with *S. gordonii* (Figure 2, Figure 7 and Figure 8). Additional analyses revealed that this phenotype is highly correlated with elevated *radD* transcript levels (Figure 3). We previously identified FAD-I as a small lipoprotein with a subspecies-dependent differential ability to induce human β-defensin 2 (hBD-2) in oral epithelial cells [21,22]. In contrast to its function in hBD-2 induction, which is only triggered by FAD-I of *F. nucleatum* subspecies *nucleatum* but not the subspecies *polymorphum*, we found that the FAD-I regulatory effect on *radD* expression and role in biofilm formation with its partner species *S. gordonii* is similar in both *F. nucleatum* subspecies tested in this study (Figure 2 and Figure 3). Hence, after establishing this similarity during the initial characterization, we focused on *F. nucleatum* ssp *nucleatum* ATCC 23726 and its mutant derivatives for more detailed investigation. We revealed that while FAD-I is required for the regulatory effect on *radD* expression, a functional RadD adhesin is not (Figure 4 and Figure 5, Supplemental Figure S1), suggesting an independent pathway for FAD-I action. Interestingly, binding to the partner species *S. gordonii* also has a regulatory impact and seems to suppress *radD* transcription largely independent of FAD-I function (Figure 6), indicating a multifactorial regulation of *radD* expression.

FAD-I is a lipoprotein, and lipoproteins in bacteria are well documented to be involved in a wide variety of functions ranging from adhesion to virulence [33,34,35]. We previously demonstrated that another small fusobacterial lipoprotein, Aid1, is involved in establishing the initial contact with streptococcal binding partners [27]. As lack of Aid1 results in reduced binding, while overexpression significantly increases attachment to streptococci and alters biofilm architecture, a direct role in fusobacterial interspecies interaction was proposed. In contrast, lack of FAD-I significantly enhances attachment to *S. gordonii*, which makes a direct role of FAD-I in the binding process unlikely. Roles in transcriptional regulation similar to what we found in our study for the effect of FAD-I on *radD* transcriptional levels have not been previously described for lipoproteins. As FAD-I is a classical small membrane-associated lipoprotein that lacks any possible DNA-binding motif [22], direct interaction with the promoter driving *radD* expression is unlikely. Likewise, RapA, which was recently described as a member of the FadA family [23] based on its similarity to FadA, a fusobacterial adhesin for binding to eukaryotic cells [18], as well as the still uncharacterized RapB do not contain any discernible DNA-binding domain. Therefore, the FAD-I-dependent regulation of *radD* expression is indirect and the connecting pathways and regulators remain to be discovered.

Regulation of adhesins is important for microorganisms as they have a central role in coordinating the development of multispecies biofilms. This regulation is therefore critical for the versatility of biofilms to respond to both harmful and beneficial factors affecting the polymicrobial community. Previous studies of *F. nucleatum* report strain-dependent variation in the adherence properties to host cells and proteins [36]. This difference in adherence capabilities and the complexity of regulatory mechanisms of adhesins likely reflect the importance of specific expression of adhesins for the survival of bacteria in changing environments. In addition to varying specificities, these differences in attachment are a result of the regulation of adhesins, reflecting the importance of adhesin regulation for the survival of bacteria in changing environments [37,38,39]. To date, there have been few reports of lipoproteins playing roles in adhesion. In addition to our previous characterization of Aid1 [27], which appears to have a direct role in interspecies binding, the lipoprotein SadB enhances the surface display of the trimeric autotransporter protein SadA in *Salmonella* and related enterobacteria [40]. Contrary to their observation of a direct role in outer membrane protein display for a lipoprotein, we found that the FAD-I lipoprotein regulates RadD on the level of transcription as the absence of FAD-I resulted in increased expression of *radD*. Enhanced expression of *radD* not only led to increased coaggregation of Δ*fad-I F. nucleatum* derivatives with *S. gordonii*, but also significantly enhanced dual-species biofilm formation compared to wild-type. The substantial increase in interspecies binding requires the presence of a functional RadD adhesin as a mutant lacking both FAD-I and RadD is unable to bind to streptococcal partner species (Figure 5). Our findings here support an important biological role for tight multi-level regulation of RadD production, since deregulated *radD* expression severely alters interspecies interaction and biofilm architecture. The modulation of RadD mediated interactions by Aid1 [27] and the results presented in this study demonstrating an effect of FAD-I, as well as binding to *S. gordonii* on transcriptional regulation of *radD*, suggest that the fine-tuning of interactions involving RadD is essential for maintaining specific interspecies interactions in the oral community.

Our observation that *radD* expression is suppressed for wild-type *F. nucleatum* spp. *nucleatum* as well as its mutant derivatives Δ*fad-I* and Δ*radD* when it is co-incubated with *S. gordonii* (Figure 6). It is consistent with other studies demonstrating that the expression of adhesin genes is downregulated once attachment is established between bacterial species. Miller et al. [41] reported similar findings for *P. gingivalis* during interaction with *Acinetobacter baumannii* where the *fim*A and *Hag*A adhesins were downregulated, presumably to conserve energy. Mostly, adhesins are regulated by a number of environmental factors along with the presence or absence of partner species. This has been well documented for the *fimA* adhesin of *P. gingivalis* [42,43,44].

In the present study, we provide evidence that lack of *fad-I* results in increased coaggregation and biofilm formation with *S. gordonii*. These findings suggest that the FAD-I protein plays an important role in the regulation of the RadD adhesin, however, additional studies are needed to further decode the mechanism of this regulation. Further identification of regulatory mechanisms of fusobacterial adhesins is necessary and will continue to clarify the central role of *F. nucleatum* interspecies interaction in health and disease.

## Figures and Tables

**Table 1:** Bacterial strains and plasmids used in the study.

Bacterial Species	Strains	Characteristics	Source
***F. Nucleatum***	**ATCC 23726**	**Ssp *Nucleatum* WT**	**ATCC**
	Fnn_Δ*rapA*	ATCC 23726 *rapA::catP*	This study
	Fnn_Δ*rapB*	ATCC 23726 *rapB:: catP*	This study
	Fnn_Δ*fad-I*	ATCC 23726 *fad-I:: catP*	This study
	Fnn_Δ*radD*	ATCC 23726::pIP1526	[15]
	Fnn_WT_CIC	ATCC 23726 *catP* inserted after *fad_I*	This study
	Fnn_Δ*fad-I*/pBS5	Δ*fad-I* 23726::pBS5	This study
	Fnn_Δfad-I radD*	ATCC 23726 Δfad-I radD*	This Study
***F. Nucleatum***	**ATCC 10953**	**Ssp *Polymorphum* WT**	**ATCC**
	Fnp_Δ*rapA*	ATCC 10953 *rapA:: catP*	This study
	Fnp_Δ*rapB*	ATCC 10953 *rapB:: catP*	This study
	Fnp_Δ*fad-I*	ATCC 10953 *fad-I:: catP*	[22]
	Fnp_Δ*radD*	ATCC 10953::pBS24	[25]
	Fnp_WT_CIC	ATCC 10953 *catP* inserted after *fad-I*	This study
	Fnp_Δfad-I*	ATCC 10953 Δfad-I*:: catP	This study
**OTHERS**			
*Escherichia Coli*	DH5α	One Shot TOP10	Invitrogen
*Streptococcus Gordonii*	Challis DL1	*attB::mCherry*	[24]
**PLASMIDS**	**Purpose**	**Characteristics**	**Source**
pBS5	Shuttle vector	*ermB*	[22]
pHS31	Suicide vector	*catP*	[26]
pHS58	Shuttle vector	*ermB*	[27]

* represents mutation in the start codon that abolishes translation into a protein.

**Table 2:** List of qPCR primers used in the study.

Primer	Targeted Locus	Primer Sequence (5′ to 3′)
BS1035 BS1036	*rapA*	GGCAAGTGATGAAATTATTTCAGAGTTAAAAGG GCTTTTAATTCAGCCAGTTTAATATTTTGAGCTG
BS1037 BS1038	*rapB*	ATTATGAAGAATTAGATAAGAAAAAAGAAAAAGAAGC CATATTATCTATTTTTTCTTTTGCTTTATCTACTTTATATTTAAG
BS947 BS948	*fad-I*	TCAAGACTTTTAAAAGAAGCTGATAAGAAAAAAG TTATTTCCCCTCTTGTCATTCCTTTATGTG
BS1066 BS1067	*radD*	GGATTTATCTTTGCTAATTGGGGAAATTATAG ACTATTCCATATTCTCCATAATATTTCCCATTAGA
BS945 BS946	*rpoB*	CAAAAACTCATTGAAAGACTTGATTTTGGA GAATGCTAATTCAAATCCTTTTTCTTCCCT

## Supplementary Materials

The following are available online at https://www.mdpi.com/2076-2607/8/1/70/s1: Figure S1: Analysis of the Δ*fad-I* mutant strain. Figure S2: Characterization of the *fad-I* translation start site mutant in *F. nucleatum* ssp *polymorphum* ATCC 10953. Table S1: List of primers used in the study for inactivation of the “*radD*” operon genes.
