# Correction: Shokeen, B., et al. Role of FAD-I in Fusobacterial Interspecies Interaction and Biofilm Formation. *Microorganisms* 2020, 8, 70

**DOI:** 10.3390/microorganisms9010063

**Published:** 2020-12-29

**Authors:** Bhumika Shokeen, Jane Park, Emily Duong, Sonam Rambhia, Manash Paul, Aaron Weinberg, Wenyuan Shi, Renate Lux

**Affiliations:** 1 Section of Periodontics, Division of Constitutive & Regenerative Sciences, UCLA School of Dentistry, Los Angeles, CA 90095, USA; bhumikas@ucla.edu (B.S.); janexpark@gmail.com (J.P.); eqduong@ucla.edu (E.D.); sonamrambhia111@gmail.com (S.R.); 2 David Geffen School of Medicine, UCLA, Los Angeles, CA 90095, USA; MPaul@mednet.ucla.edu; 3 Department of Biological Sciences, Case Western Reserve University, Cleveland, OH 44106-4905, USA; axw47@case.edu; 4 The Forsyth Institute, Cambridge, MA 02142, USA; wshi@forsyth.org

The authors wish to make the following corrections to this paper [1]:

In the Materials and Methods Section 2.2.1, part of the method was incorrect and instead of

“The plasmid DNA of the recombinants were isolated with the Qiagen Mini prep kit (Qiagen, Hilden, Germany) and the presence of construct was confirmed by restriction digestion and sequencing. The construct was further subcloned in the suicide vector pHS31 as follows: both the fusion construct and pHS31 were digested with EcoRI/BamHI and purified prior to ligation (Supplementary Figure S1) and transformation into *Escherichia coli*. After confirmation of the integration plasmid by restriction analysis and sequencing, purified plasmids were electroporated into the fusobacterial strains used in this study to generate the respective derivatives lacking target genes according to previously described protocols [29]”.

The method should be:

“The plasmid DNA of the recombinants were isolated with the Qiagen Mini prep kit (Qiagen, Hilden, Germany) and the presence of the construct was confirmed by restriction digestion and sequencing. Further, purified plasmids were electroporated into the fusobacterial strains used in this study to generate the respective derivatives lacking target genes according to previously described protocols (Supplementary Figure S1) [29]”.

The authors would like to apologize for any inconvenience caused to the readers by these changes.

## Data Availability

The strains and plasmids used in this study are available on request from the corresponding author.
