# Supplementary material for: Role of FAD-I in Fusobacterial Interspecies Interaction and Biofilm Formation
Source: Microorganisms. 2020 Jan 2;8(1):70. doi: 10.3390/microorganisms8010070 (PMC7023056; doi:10.3390/microorganisms8010070)
Supplement: Supplementary file 1 [file microorganisms-08-00070-s001.pdf]

Supplementary Table 1: List of primers used in the study for inactivation of the "*radD*" operon genes

Strain	Primer	Sequence (5′ to 3′)
Fnp_Δ*rapA*	FnpA_upF	TTACATGGGGTGGAGGAATCTTCTTAGC
	FnpA _upR	ATCGATCCCCGCCGAGCGAAACTCACCTCTCCTTTAATTTCAATAAAATATATAGTATAA
	FnpA_catPF	GAAATTAAAGGAGAGGTGAGTTTCGCTCGGCGGGGATCGAT
	FnpA_catPR	CTTTTATTTTCATTTTTCCCCCTCATTATTAACTATTTATCAATTCCTGCAATTCG
	FnpA_DnF	CGAATTGCAGGAATTGATAAATAGTTAATAATGAGGGGGAAAAATGAAAATAAAAGAAAT
	FnpA_DnR	TTATTTCTGTTCTTAATGGCACTTGTATTGC
Fnp_Δ*rapB*	FnpB_upF	CTATGATGCAATATAAGTTCTCCTTTAATAACCTTAAATATAC
	FnpB_upR	ATCGATCCCCGCCGAGCGTTTTCCCCCTCACTATCTTATTTTTTGAATTTTC
	FnpB_catPF	TAAGATAGTGAGGGGGAAAACGCTCGGCGGGGATCGAT
	FnpB_catPR	CTTTTTCAAAATTTTCCCCTCCCTTTATTAACTATTTATCAATTCCTGCAATTCG
	FnpB_DnF	CGAATTGCAGGAATTGATAAATAGTTAATAAAGGGAGGGGAAAATTTTGAAAAAG
	FnpB_DnR	GGTGTTACCCTTGGTGCTTCTATTATCTTTTG
Fnp_Δ*fad-I**	FnpC*_F	GGAGGGGAAAATTT**AAT**AAAAGATATTACTACTATTATTATC
	FnpC*_R	CTTTATTTTTCTTCTGTAATATTTTTTAAAGCTTCTTCAACTTG
Fnp_WT_CIC	FnpCIC_upF	GCAGAATATGAAGATCTAGTAAAAGAAGAAGAAGC
	FnpCIC_upR	TTATTTTATTCCTGCATTATTTAATTCTTCTAATTTTTG
	FnpCIC_catPF	CGCTCGGCGGGGATCGAT
	FnpCIC_catPR	TTAACTATTTATCAATTCCTGCAATTCG
	FnpCIC_DnF	TAAGAGGGGGGGAAAATATGAAAGACT
	FnpCIC_DnR	AATTGAGATATCAATCCATTATTTCCAGTTAC
Fnn_Δ*fad-IradD**	FnnCD*_upF	GGCGCTGGTACCACTAATAATTTTATATTTCGAGAGACAAAAGCATT
	FnnCD*_upR	ATCGATCCCCGCCGAGCGCAAATTTTTTCCCTCCCTTTATTTTTCT
	FnnCD*_catPF	AGAAAAATAAAGGGAGGGAAAAAATTTGCGCTCGGCGGGGATCGAT
	FnnCD*_catPR	ACTTTATTATAGTCTTCATATTTTCCCCCTCTTATTAACTATTTATCAATTCCTGCAATTCG
	FnnCD*_DnF	CGAATTGCAGGAATTGATAAATAGTTAATAAGAGGGGGAAAATATGAAGACTATAATAAAGT
	FnnCD*_DnR	GGCCGAGCTCGAGTGGTGTAAAACCTGCTGGTGTAGCA
Fnn_Δ*rapA*	FnnA_upF	GAGAAAATAAAATTGAAATA
	FnnA_upR	ATCCCCGCCGAGCGAAATATTCCAATAGATAATAAAACAAATAATGTTAAAATAACTTT
	FnnA_catPF	GTTTTATTATCTATTGGAATATTTCGCTCGGCGGGGATCG
	FnnA_catPR	TTAACTATTTATCAATTCCTGCA
	FnnA_DnF	GGAATTGATAAATAGTTAATGAGGGGGAAAAATGAAAATAAAAGAAAT
	FnnA_DnR	CTTGTCTTTATTTCTGTTCTTAATGGCACTTG
Fnn_Δ*rapB*	FnnB_upF	CTGTTGCTATTGATATTGGTTTCCCAGC
	FnnB_upR	CGATCCCCGCCGAGCGTTTTCCCCCTCACTATCTTATTTTTTGAATT
	FnnB_catPF	AATTCAAAAAATAAGATAGTGAGGGGGAAAACGCTCGGCGGGGATCG
	FnnB_catPR	CAAATTTTTTCCCTCCCTTTAACTATTTATCAATTCCTGCAATTCGTTTAC
	FnnB_DnF	GAATTGATAAATAGTTAAAGGGAGGGAAAAAATTTGAAAAAAATATTATTAC
	FnnB_DnR	CTGTTTTTTCAATTATTGTTTTTTCAATTACTGC

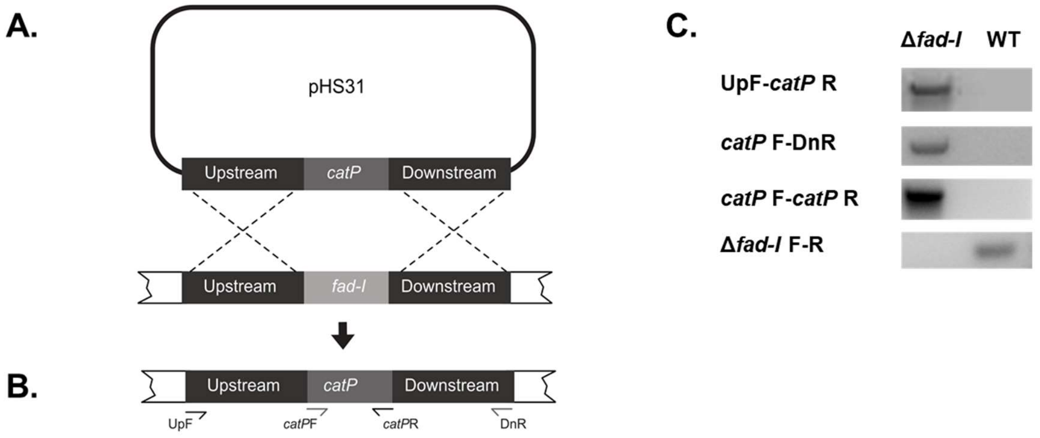

Supplementary Figure S1: Analysis of the Δ*fad-I* mutant strain. **A.)** Diagram depicting the allelic exchange mutagenesis using suicide vector pHS31 with the construct for inactivation of *fad-I*. This plasmid was used for transformation in *F. nucleatum ssp nucleatum* 23726 to generate the Fnn_Δ*fad-I* mutant **B.)** Schematic representation of the Δ*fad-I* mutant after transformation with the suicide vector pHS31 with the construct. The arrows indicate the location of the primers used for PCR amplification. **C.)** Confirmation of the Δ*fad-I* mutant using various internal primers of the construct. The internal primers of the construct amplified fragments of the expected size in the mutant strain but not in the wild-type control. The absence of the *fad-I* gene was further confirmed by its absence in the Δ*fad-I* mutant and presence in the wild type control.

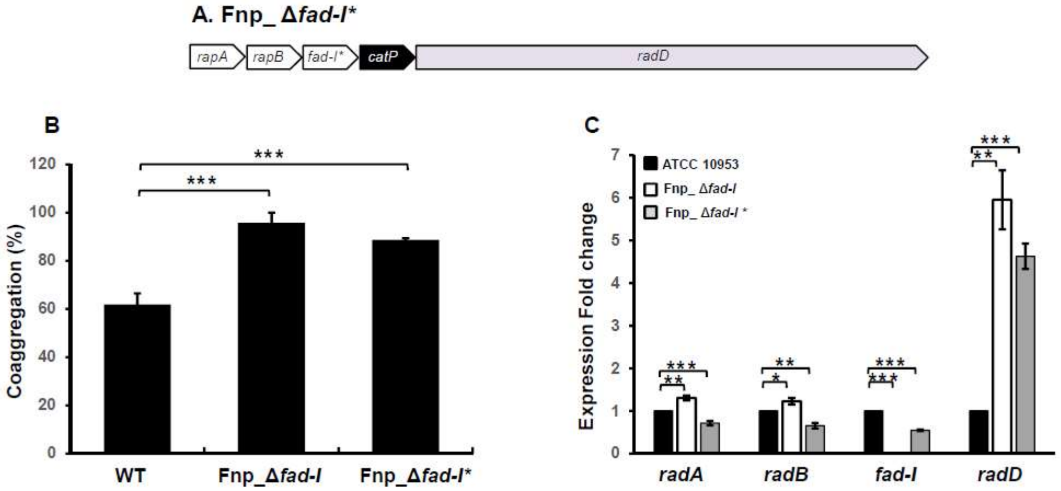

Supplementary Figure S2: Characterization of the *fad-I* translation start site mutant in *F. nucleatum* ssp *polymorphum* ATCC 10953 (A) Schematic representation of Fnp_Δ*fad-I** **(B)** coaggregation of Fnp_Δ*fad-I** with *S. gordonii* is represented as mean of percentage coaggregation *along* with WT and Fnp_Δ*fad-I* **(C)** expression fold-change of *rapA, rapB, fad-I* and *radD* in Fnp_Δ*fad*-I* and Fnp_Δ*fad*-I compared to the wild type.
